# LINC02595 promotes tumor progression in colorectal cancer by inhibiting miR‐203b‐3p activity and facilitating BCL2L1 expression

**DOI:** 10.1002/jcp.29650

**Published:** 2020-02-16

**Authors:** Zhidong Yang, Yue An, Ningning Wang, Xihua Dong, Hui Kang

**Affiliations:** ^1^ Department of Laboratory Medicine The First Affiliated Hospital of China Medical University Shenyang Liaoning China; ^2^ Department of Gastroenterology The First Affiliated Hospital of China Medical University Shenyang Liaoning China

**Keywords:** apoptosis, cell cycle, colorectal cancer, LINC02595, miR‐203b‐3p

## Abstract

Colorectal cancer (CRC) is one of the most prevalent tumors worldwide. Recently, long noncoding RNAs (lncRNAs) have been recognized as key regulators in postgenomic biology. Numerous lncRNAs have been identified as diagnostic biomarkers and therapeutic targets. However, the molecular mechanisms underlying the role of lncRNAs in CRC progression are not fully understood. Differentially expressed lncRNAs and messenger RNAs were investigated using a microarray approach in five paired primary CRC tumor tissues and the corresponding nontumor tissues and confirmed in an additional 116 paired tissues and 21 inflammatory bowel disease tissues and 15 adjacent normal tissues by a quantitative real‐time polymerase chain reaction. We also performed comprehensive transcriptome profiling analysis on Gene Expression Omnibus and The Cancer Genome Atlas datasets. We identified LINC02595 and evaluated its clinical significance as a plasma biomarker. The function of LINC02595 was evaluated using a panel of in vivo and vitro assays, including cell counting kit‐8, colony formation, cell cycle, apoptosis, RNA fluorescence in situ hybridization, luciferase reporter, immunohistochemistry, and CRC xenografts. We found that LINC02595 is upregulated in tumor tissues and blood samples of patients with CRC and CRC cell lines. Functional research found that LINC02595 promotes CRC cell growth, influences the cell cycle, and reduces apoptosis in vitro and vivo. Mechanistically, LINC02595 promoted BCL2‐like 1 (BCL2L1) expression through miR‐203b‐3p sponging. Our research demonstrated that LINC02595 is an oncogene in CRC and established the presence of a LINC02595‐miR‐203b‐BCL2L1 axis in CRC, which might provide a new diagnostic biomarker and therapeutic targets for the treatment of this disease.

## Graphical abstract

Our research demonstrated that LINC02595 is an oncogene in colorectal cancer (CRC) and established the presence of a LINC02595‐miR‐203b‐BCL2L1 axis in CRC, which might provide a new diagnostic biomarker and therapeutic targets for the treatment of this disease.

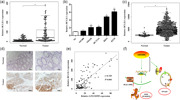

## 1 INTRODUCTION

Colorectal cancer (CRC) is one of the three most common cancers worldwide (Siegel et al., 2017). Although there have been improvements in the treatment of this disease, most patients with CRC are still diagnosed with advanced‐stage disease because of a shortage of useful biomarkers (Sepulveda et al., 2017). Advanced‐stage disease with distant metastasis is the main reason for the poor therapeutic efficacy of surgery. Therefore, discovering highly effective diagnostic biomarkers and understanding the molecular mechanisms underlying CRC tumorigenesis are needed to significantly increase the survival rate of patients with CRC.

Long noncoding RNAs (lncRNAs) are endogenous noncoding RNAs with transcripts longer than 200 nucleotides (Uszczynska‐Ratajczak, Lagarde, Frankish, Guigo, & Johnson, 2018). An increasing number of studies have shown that lncRNAs are involved in many diseases, including CRC (Dong et al., 2019; Lee et al., 2019; Wang et al., 2019). They play a pivotal role in modulating tumor progression and may also be used as biomarkers for a variety of cancers. Numerous lncRNAs are abnormally expressed in CRC, including H19 (Ding et al., 2018), PVT1 (He et al., 2019), SNHG6 (Xu et al., 2019), SNHG1 (Bai, Xu, Zhao, & Zhang, 2019), CCAT (Y. Li et al., 2018), and MALAT1 (Zhuang et al., 2019). These lncRNAs act as competing endogenous RNAs (ceRNAs) to exert their functions. However, the mechanisms of most lncRNAs in CRC remain unknown.

In the present study, our objective was to identify novel lncRNAs in CRC through a comprehensive analysis of microarray, The Cancer Genome Atlas (TCGA), and Gene Expression Omnibus (GEO) datasets. We determined the lncRNA expression profile in primary tumor tissue and paired adjacent normal tissue using the lncRNA and messenger RNA (mRNA) microarray. Through a detailed analysis of the expression of lncRNAs in various tissues by quantitative real‐time polymerase chain reaction (qRT‐PCR) and verification in TCGA and GEO datasets, we found that LINC02595 was significantly upregulated in CRC tissues. We further evaluated the function of LINC02595 through bioinformatics and experimentally in CRC cells. Moreover, we established LINC02595 as a novel biomarker and oncogene that functions as a ceRNA of miR‐203b‐3p to regulate the expression of BCL2‐like 1 (BCL2L1) in CRC. This study identified a potential new biomarker and therapeutic target for CRC.

## 2 MATERIALS AND METHODS

### 2.1 Microarray and computational analysis

The samples (five colorectal primer tumor tissues and their adjacent nontumor tissues) were used to purify RNA and synthesize double‐stranded complementary DNA (cDNA), which was hybridized to the Human LncRNA Gene Expression Microarray V4 (CapitalBio Corp, Beijing, China). The lncRNA and mRNA array data were subjected to data summarization, normalization, and quality control using GeneSpring software V13.0 (Agilent). A fold‐change (FC) of >2 and a threshold of *p* < .05 were used to select differentially expressed lncRNAs.

### 2.2 Expression profile analysis from GEO and TCGA

Three CRC raw microarray datasets (GSE110715, GSE109454, and GSE70880) were downloaded from the GEO database. Raw data for 506 patients with CRC were downloaded from TCGA data portal. Perl and R package were used to analyze all the datasets with FC > 2 and *p* < .05 as a threshold.

### 2.3 Gene set enrichment analysis

Gene set enrichment analysis v3.0 (GSEA v3.0) was carried out using the JAVA program, and enrichment analysis were performed on normalized mRNA expression profiles. Random sample permutations (*n* = 1,000) were used to calculate the *p* value. A false discovery rate (FDR) <0.01 and a nominal *p* < .05 were considered as significant.

### 2.4 Tissue samples and plasma samples

Paired fresh CRC and adjacent nontumor tissues were collected from patients (*n* = 116), who underwent surgery without preoperative anticancer treatment at The First Affiliated Hospital of China Medical University in 2017. Detailed clinicopathological characteristics were recorded. All specimens were rapidly frozen in liquid nitrogen for 24 hr and then stored at −80°C until RNA extraction. The study protocol was approved by the Research Ethics Committee of The First Affiliated Hospital of China Medical University. All individual patients provided signed informed consent. Plasma samples from 30 patients with CRC and 25 healthy donors of matched age and gender were obtained before surgery between 2017 and 2019. The plasma samples were stored at −80°C until further analysis.

### 2.5 RNA extraction and qRT‐PCR analysis

Total RNA was isolated from tissues and cells using TRIzol reagent (Invitrogen, Carlsbad) following the manufacturer's instructions. For plasma samples, total RNA was isolated from cell‐free plasma (200 µl) using a miRNeasy Serum/Plasma Kit (Qiagen, Germany) following the manufacturer's instructions. cDNA was synthesized using a Transcriptor First‐Strand cDNA Synthesis Kit (Roche) following the manufacturer's instructions.

qRT‐PCR was performed using a FastStart Universal SYBR Green Master (ROX; Roche) and a LightCycler 480. Relative expression levels were normalized to glyceraldehyde‐3‐phosphate dehydrogenase (GAPDH) or U6 expression. The primers used for target amplification are presented in Table 1. All qRT‐PCR results were analyzed using $2^{-\Delta \Delta C_{t}}$ the values.

**Table 1:** The sequences of the PCR primers used in present study

Gene	Sequence
LINC02595	Forward: 5′ ‐ GTGGCAGTGTAGAAGCCAGAC‐ 3′
	Reverse: 5′ ‐ GGCAAATTAGTGGGCCTGTGG‐ 3′
GAPDH	Forward: 5′ ‐ GCACCGTCAAGGCTGAGAAC‐ 3′
	Reverse: 5′ ‐ TGGTGAAGACGCCAGTGGA‐ 3′
miR‐203b‐3p	Forward: 5′ ‐ GCGCGTTGAACTGTTAAGAACC‐ 3′
	Reverse: 5′ ‐ AGTGCAGGGTCCGAGGTATT‐ 3′
miR‐3942–3p	Forward: 5′ ‐ CGCGCGTTTCAGATAACAGTA‐ 3′
	Reverse: 5′ ‐ AGTGCAGGGTCCGAGGTATT‐ 3′
miR‐4715–3p	Forward: 5′ ‐ CGGTGCCACCTTAACTGCA ‐ 3′
	Reverse: 5′ ‐ AGTGCAGGGTCCGAGGTATT‐ 3′
U6	Forward: 5′ ‐ AGAGAAGATTAGCATGGCCCCTG‐ 3′
	Reverse: 5′ ‐ AGTGCAGGGTCCGAGGTATT‐ 3′
BCL2L1	Forward: 5′ ‐ CGTGGAAAGCGTAGACAAG‐ 3′
	Reverse: 5′ ‐ AAGAGTGAGCCCAGCAGAA‐ 3′

Abbreviations: BCL2L1, BCL2‐like 1; GAPDH, glyceraldehyde 3‐phosphate dehydrogenase; miR, microRNA; PCR, polymerase chain reaction.

### 2.6 Cell lines and culture

Six human CRC cell lines (SW620, HT29, HCT116, RKO, Caco‐2, and SW480) and human embryonic kidney (HEK) 293T cells were purchased from the Cell Bank of Type Culture Collection of the Chinese Academy of Sciences (Shanghai, China). SW620, SW480, HCT116, HT29, and RKO cells were maintained in Roswell Park Memorial Institute 1640 (HyClone, Logan, UT), Caco‐2 and 293T were maintained in Dulbecco's modified Eagle's medium containing high glucose (HyClone, Logan, UT). All media were supplemented with 10% fetal bovine serum (Gibco, Grand Island, NY). The cells were grown in a humidified 5% CO_2_ incubator at 37°C.

### 2.7 Subcellular fractionation

The cytoplasm and nuclear fractions of cells were separated using the PARIS Kit (Invitrogen) following the manufacturer's instructions. qRT‐PCR was used to analyze the ratios of expression, and the results were calculated using the $2^{-\Delta \Delta C_{t}}$ values. U6 and GAPDH served as the nuclear and cytoplasmic markers, respectively.

### 2.8 RNA fluorescence in situ hybridization

Cy3‐labeled LINC02595 probes (RiboBio, Guangzhou, China) were used for fluorescence in situ hybridization (FISH) assays in RKO and HT29 cells, according to the manufacturer's protocol.

### 2.9 LINC02595 smart silencer, plasmid, and miRNA transfection

The LINC02595 smart silencer, miR‐203b‐3p, miR‐4715‐3p, miR‐3942‐3p mimics, miR‐203b‐3p inhibitor, and negative controls (NCs) were synthesized by RiboBio (Guangzhou, China). The LINC02595 smart silencer was a mixture that included three small interfering RNAs (siRNAs) and three antisense oligonucleotides. The antisense sequences of the siRNAs were: (a) GGGAGCTTCCAGAAGTGGT, (b) CAAAAGAATTTGCCTTTGA, and (c) AATAAGGACGAGTTATGTGC. Cells were transfected using Lipofectamine RNAiMAX (Invitrogen, Carlsbad) following the manufacturer's protocol. Human LINC02595 cDNA was synthesized and cloned into the pcDNA3.1 vector. Transfection of LINC02595 pcDNA3.1 was performed using Lipofectamine 2000 (Invitrogen, Carlsbad) according to the manufacturer's protocol.

### 2.10 Stable LIN02595 knockdown cell lines

HT29 cells were transfected with lentiviruses expressing sh‐LINC02595 or sh‐empty vector in the presence of 5 μg/ml polybrene for 24 hr followed by puromycin (4 μg/ml) selection for 5 days. LINC02595 expression was confirmed by qRT‐PCR.

### 2.11 Mouse xenografts

The mouse experiments were approved by the Institutional Ethics Committee of China Medical University. Ten BALB/c immunodeficient mice 4–6 weeks of age) were inoculated subcutaneously with stable HT29‐sh‐empty vector or HT29‐sh‐LINC02595 cells (4 × 10^6^ in 0.2 ml) into the mouse abdomen. Tumor volumes were measured as (length × width^2^)/2 every 5 days. All the mice were euthanized 20 days postinoculation.

### 2.12 Cell proliferation

CRC cell proliferation was measured using the cell counting kit‐8 assay (Dojindo Lab, Japan) following the manufacturer's protocol. Cell proliferation was also evaluated using the colony formation assay. CRC cells (1 × 10^3^/well) were seeded into six‐well plates. After incubation for 10 days, the cells were fixed with formaldehyde, stained with 0.5% crystal violet, rinsed with H_2_O, and air‐dried. Colonies were counted using ImageJ software.

### 2.13 Flow cytometry

Apoptosis was measured using the FITC Annexin V Apoptosis Detection Kit (BD Biosciences) following the manufacturer's instructions. The cell cycle was analyzed using the PI/RNase Staining Buffer Solution Kit (BD Biosciences) following the manufacturer's instructions.

### 2.14 Dual‐luciferase reporter assay

For the LINC02595 promoter‐luciferase reporter assay, HEK293T cells were cotransfected with the pmirGLO report vector carrying wild‐type (WT) LINC02595 or mutant‐type (MT) LINC02595 and miR‐203b‐3p, miR‐4715‐3p, miR‐3942‐3p mimics or miR‐NC using Lipofectamine 2000 (Invitrogen). For the miR‐203b‐3p target gene BCL2L1 luciferase reporter assay, HEK293T cells were cotransfected with the pmirGLO report vector carrying WT‐BCL2L1 or MT‐BCL2L1 and miR‐203b‐3p mimics or miR‐NC using Lipofectamine 2000 (Invitrogen). Forty‐eight hours after transfection, luciferase activity was measured using the Dual‐Luciferase Reporter Assay Kit (Promega, Madison) according to the manufacturer's instructions.

### 2.15 Immunohistochemistry

Tissue samples were fixed in 10% formalin following general instructions. For hematoxylin and eosin (HE) staining, paraffin‐embedded tissues were dewaxed, rehydrated, HE stained, and dehydrated. For immunohistochemistry (IHC), UltraSensitive^TM^ SP (Mouse/Rabbit) IHC Kit (MXB Biotechnologies, Fuzhou, China) and DAB Kit (20X; MXB Biotechnologies, Fuzhou, China) were used according to the manufacturer's instructions.

### 2.16 Western blot analysis

Total protein was isolated from cells using radioimmunoprecipitation assay lysis buffer. The protein concentration of each sample was measured using the BCA Protein Assay Kit (Beyotime, Shanghai, China). Equal amounts of protein were separated by 12% sodium dodecyl sulfate–polyacrylamide gel electrophoresis and electro‐transferred to 0.2 µm polyvinylidene fluoride membranes. The blots were blocked with 5% skimmed milk in Tris‐buffered saline (TBS) at 37°C for 2 hr and then incubated with primary antibodies overnight at 4°C: BCL2L1 (1:1,000; Cell Signaling Technology), Bcl2 (1:1,000, Wanleibio, Beijing, China), BAX (1:1,000; Wanleibio, Wanleibio, Beijing), CDK4 (1:1,000; Cell Signaling Technology), CDK6 (1:1,000; Cell Signaling Technology), Survivin (1:1,000; Cell Signaling Technology), and β‐tubulin (1:2,000; Wanleibio, Beijing, China). The membranes were washed three times with TBS with Tween‐20 and then incubated with secondary antibody (1:5,000; Abbkine, CA) for 1 hr at room temperature. Protein bands were detected using BeyoECL Plus kit (Beyotime, Shanghai, China).

### 2.17 Statistical analyses

All statistical analyses were performed using SPSS 22.0 software (IBM). Significance between groups was determined using Student's *t* test or Wilcoxon's test. Kaplan–Meier analysis was used to determine the overall survival (OS). Pearson's correlation analysis was used to calculate the correlation between LINC02595, miR‐203b‐3p, and BCL2L1 expression. *p* < .05 was considered statistically significant.

## 3 RESULTS

### 3.1 Comprehensive analysis of lncRNAs expression profiles in CRC using lncRNA microarray, TCGA transcriptome profiling, and GEO microarray datasets

**Figure 1:**
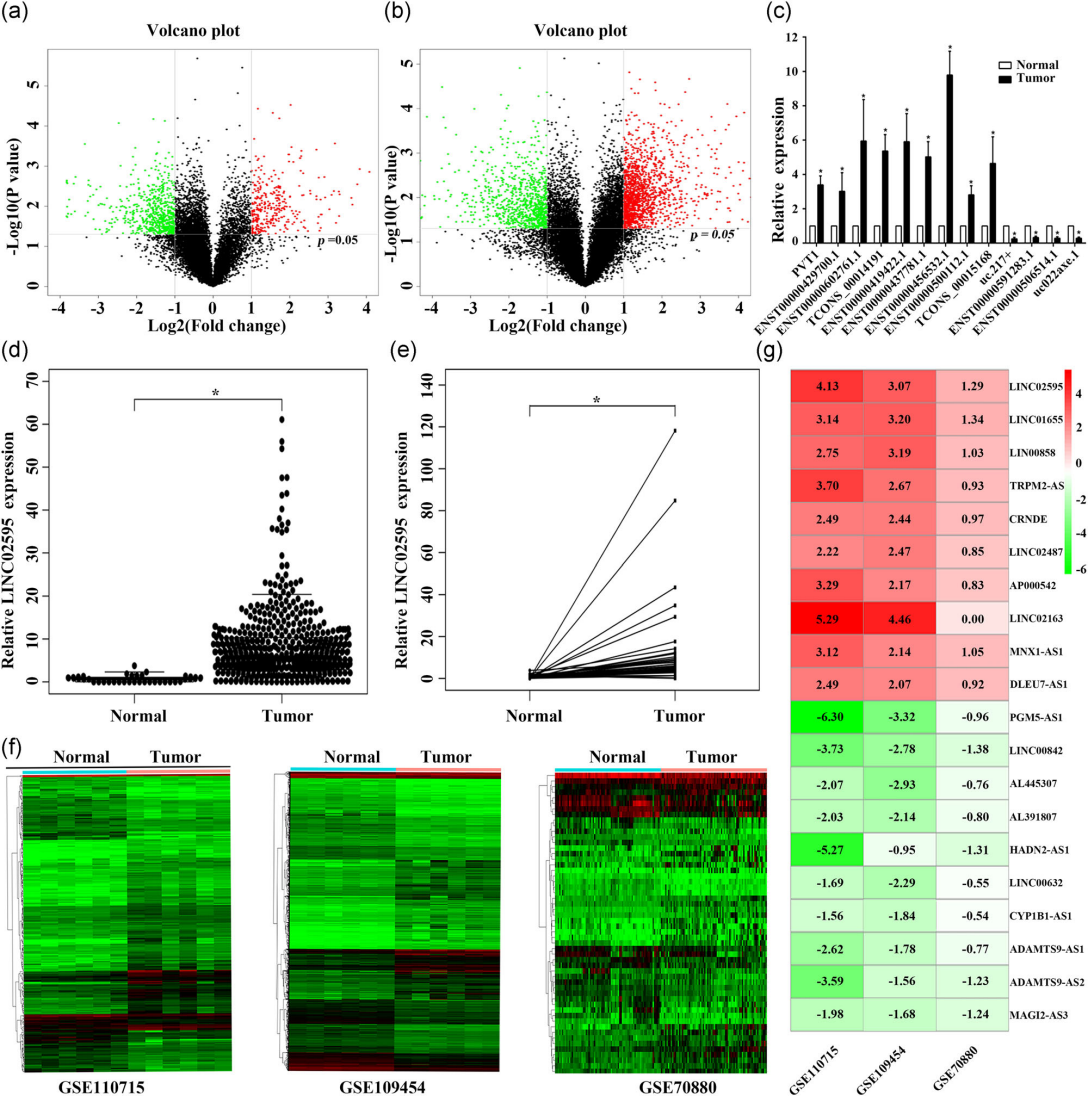
lncRNAs expression profiling in CRC tissues. (a) The volcano plot of lncRNA expression from The Human LncRNA Gene Expression Microarray V4. The upregulated lncRNAs are shown as red spots, and downregulated lncRNAs are shown as green spots (FC > 2; *p* < .05). (b) The volcano plot of mRNA expression from The Human LncRNA Gene Expression Microarray V4. The upregulated mRNAs are shown as red spots, and downregulated mRNAs are shown as green spots (FC > 2; *p* < .05). (c) Differential expression levels of 12 lncRNAs in 10 pairs of CRC tissues and corresponding adjacent normal tissues. *p < .05. (d) Data mining of LINC02595 expression in TCGA CRC tissues compared with nontumor tissues. (e) Data mining of LINC02595 expression in 41 paired CRC tissues from the TCGA dataset. *p < .05. (f) The heatmaps of microarray lncRNA profiling results (GSE110715, GSE109454, GSE70880). (g) The comprehensive transcriptional analysis of three independent microarray datasets. CRC, colorectal cancer; FC, fold‐change; lncRNA, long noncoding RNA; mRNA, messenger RNA; TCGA, The Cancer Genome Atlas

The initial lncRNA and mRNA microarray analysis to identify lncRNAs and mRNAs that are dysregulated in CRC was performed using five pairs of matched CRC and adjacent non‐cancer tissue samples. We identified 236 lncRNAs and 1,500 mRNAs that were upregulated, and 620 lncRNAs and 895 mRNAs that were downregulated in the CRC tissues compared with the adjacent normal tissue (FC > 2; *p* < .05; Figure 1a,b). The first 15 upregulated lncRNAs and mRNAs, the 15 downregulated lncRNAs and mRNAs with their FC, and the *p* values are shown in Table S1,2. The microarray accession number in the National Center for Biotechnology Information Gene Expression Omnibus for this dataset is GSE134834. To validate the microarray results, we randomly selected 12 lncRNAs transcripts according to the FCs and analyzed their expression levels in another ten pairs of CRC and adjacent normal tissue. The results indicated that ENST00000429700.1, ENST00000602761.1, TCONS_00014191, ENST00000419422.1, ENST00000437781.1, ENST00000500112.1, TCONS_00015168, and ENST00000456532.1 were overexpressed in CRC tissues, whereas the expressions of uc.217+, ENST00000591283.1, ENST00000506514.1, and uc022axe.1 was decreased. We also used lncRNA PVT1, which was previously identified in CRC, as a control. The results from this additional analysis were consistent with microarray results and identified ENST00000456532.1 (LINC02595) as the most significantly overexpressed lncRNA in CRC tissues (*p* < .05; Figure 1c).

To determine if LINC02595 plays a pivotal role in the pathogenesis of CRC, we analyzed the transcriptome profiles of CRC in the TCGA dataset. A total of 506 patients with CRC were enrolled in the study, which contained 534 tumors and 41 adjacent non‐cancer tissues. Using a cutoff threshold, we identified 869 upregulated and 756 downregulated mRNAs. In addition, 823 lncRNAs were differentially expressed (logFC > 2 and FDR < 0.01), including 622 upregulated and 201 downregulated lncRNAs (Figure S1a,b). We found that LINC02595 was also significantly upregulated in 534 unpaired and 41 paired patients with CRC samples (*p* < .05; Figure 1d,e). Correlation analysis between the LINC02595 expression levels and patient clinical features did not identify any association between LINC02595 expression and gender, age, tumor location, tumor invasion depth, or distant metastasis in CRC (Table S3). Furthermore, the OS rate of patients with underexpressed LINC02595 was better than that of patients with overexpressed LINC02595. However, this difference was not statistically significant (*p* = .36; Figure S1c). Finally, a comprehensive transcriptional analysis of three independent microarray datasets (GSE110715, GSE109454, and GSE70880) from the GEO dataset confirmed that LINC02595 was upregulated in CRC tissues (*p* < .05; Figure 1f,g). Taken together, these results revealed that LINC02595 upregulation might have a critical role in the progression of CRC.

### 3.2 LINC02595 is upregulated in CRC tissues

**Figure 2:**
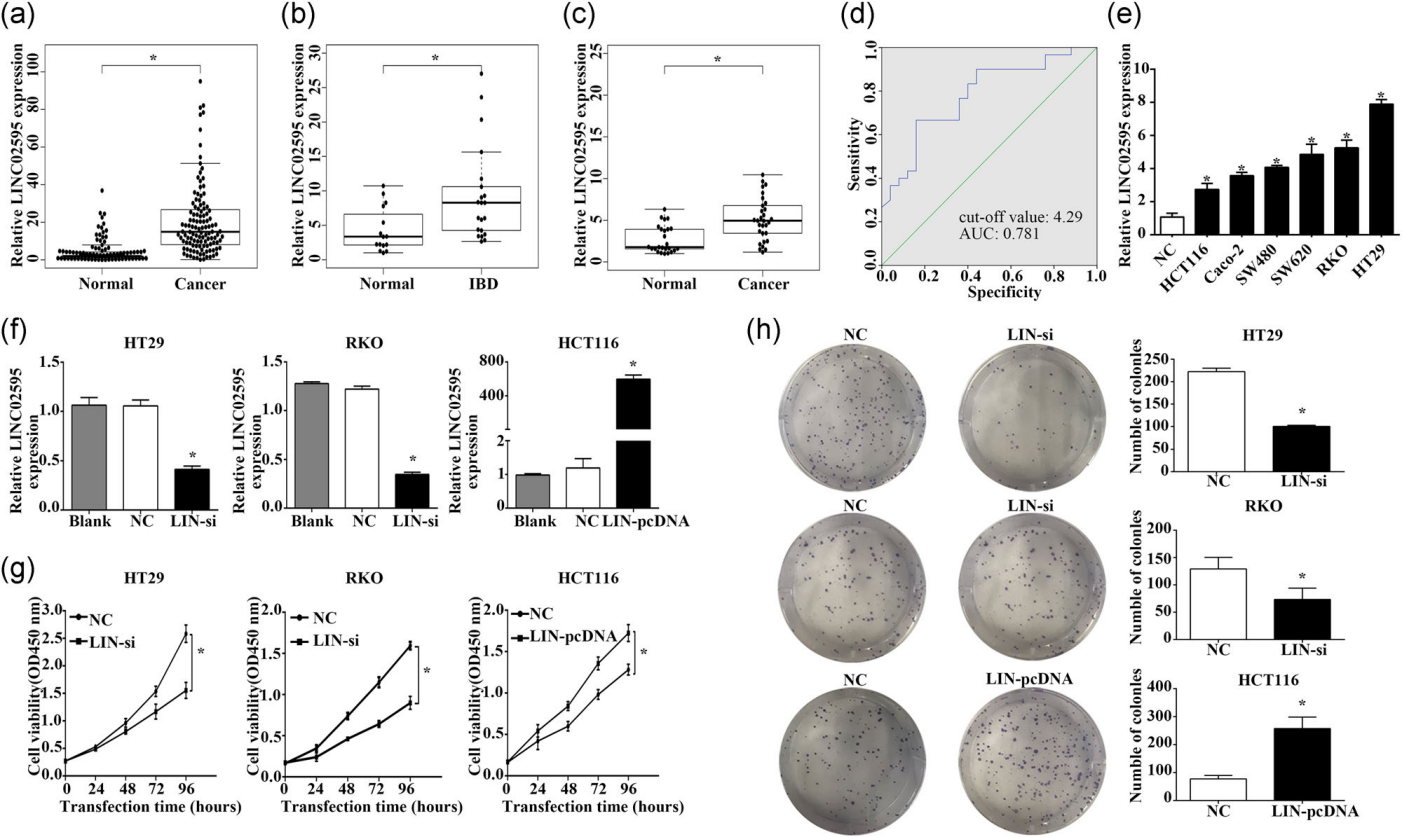
The LINC02595 expression in CRC tissues, IBD tissues, CRC plasm, CRC cells, and the impacts on proliferation of CRC cells. (a–c) The LINC02595 expression of 116 pairs of CRC and adjacent nontumor tissues (a), IBD tissues (*n* = 21) compared with non‐IBD tissues (*n* = 15) (b), and CRC plasma (*n* = 30) compared with control plasma (*n* = 26) (c). *p < .05. (d) Receiver operator curve (ROC) analysis of plasma LINC02595 in patients with CRC and control. The value of the area under the curve was 0.781 and the cut‐off value was 4.29. (e) The LINC02595 expression in six CRC cell lines compared with normal colorectal epithelial tissues. *p < .05. (f) The LINC02595 expression in HT29, RKO cells transfected with smart silencer and in HCT116 cells transfected with pcDNA. *p < .05. (g and h) Cell proliferation was investigated by (g) CCK‐8 and (h) colony formation in LIN‐si transfected HT29, RKO, and LIN‐pcDNA transfected HCT116. *p < .05. CCK‐8, cell counting kit‐8; CRC, colorectal cancer; IBD, inflammatory bowel disease

qRT‐PCR was performed on 116 pairs of CRC and adjacent nontumor tissues to confirm the bioinformatics results and understand the role of LINC02595 in the pathogenesis of the disease. LINC02595 expression was markedly upregulated in tumor tissues compared with the corresponding adjacent normal tissues (*p* = 9.78e−23; Figure 2a). The LINC02595 expression levels in the CRC samples were correlated with tumor invasion depth (*p* = .039), lymph node metastasis (*p* = .011), and distant metastasis (*p* = .019). However, no association was observed with other parameters (e.g., gender, age, and cancer subtype; Table 2).

**Table 2:** The clinical–pathological factors of 116 patients with CRC

Characteristics	Number of cases	LINC02595 expression		*p* Value^a^
		Low (*n* = 58)	High (*n* = 58)	
Age (year)				.492
<60	24	10	14	
≥60	92	48	44	
Gender				
Female	49	22	27	.452
Male	67	36	31	
Tumor invasion depth				
T1+T2	83	47	36	.039
T3+T4	33	11	22	
Lymph node metastasis				
N0	92	52	40	.011
N1+N2	24	6	18	
Distant metastasis				
M0	93	52	41	.019
M1+M2	23	6	17	
Cancer type				
Rectal cancer	96	45	51	.219
Colon cancer	20	13	7	

Abbreviation: CRC, colorectal cancer.

^a^ Statistical significant results (in italics).

There have been many studies that have demonstrated that patients with inflammatory bowel disease (IBD) have a high risk of developing CRC. Therefore, we hypothesized that LINC02595 expression might also be elevated in patients with IBD. To evaluate this hypothesis, LINC02595 expression was measured in 21 IBD and 15 adjacent normal tissues. We found that LINC02595 was overexpressed in the IBD tissues but to a lesser extent than in patients with CRC (*p* = .004; Figure 2b). These data suggest that LINC02595 could be an important marker for the early diagnosis of CRC.

### 3.3 LINC02595 is a potential diagnostic biomarker for CRC

Many studies have demonstrated that lncRNAs could represent a potential new class of diagnostic biomarkers. As LINC02595 was upregulated in CRC tumor tissues, we investigated whether LINC02595 expression was increased in peripheral blood plasma, which would make it a potential biomarker for CRC diagnosis. To this end, qRT‐PCR was performed to detect LINC02595 expression in plasma. We found that LINC02595 expression was increased in the plasma from CRC patients compared with control samples (*p* = 3.856e−04; Figure 2c). Based on the receiver operator curve (ROC) analysis between patients with CRC and controls, the cut‐off value for plasma LINC02595 was 4.29 with an area under the curve (AUC) of 0.781. The sensitivity and specificity for LINC02595 expression in the plasma were 0.667 and 0.84 (Figure 2d).

### 3.4 LINC02595 knockdown inhibits CRC cell proliferation

To investigate the biological function of LINC02595 in CRC, LINC02595 expression was measured in six CRC cell lines (HCT116, HT29, RKO, Caco‐2, SW620, SW480) by qRT‐PCR. We found that LINC02595 expression was upregulated in all six CRC cell lines compared with normal colorectal epithelial tissues (all *p* < .05; Figure 2e). The highest expression levels were observed in RKO and HT29 cells, which were selected for investigating the functional role LINC02595 in CRC cells using the transfection of a LINC02595 smart silencer. In addition, the human LINC02595 cDNA sequence was cloned to overexpress LINC02595 in HCT116 cells. The efficiencies of knockdown and overexpression were confirmed by qRT‐PCR (*p* < .05; Figure 2f).

The proliferation of HT29 and RKO cells was significantly inhibited by LINC02595 knockdown, whereas LINC02595 overexpression accelerated the proliferation of HCT116 cells (*p* < .05; Figure 2g). Furthermore, the results of colony formation confirmed that LINC02595 knockdown reduced the numbers of colony formed by HT29 and RKO cells, whereas overexpression of LINC02595 promoted the colony formation by HCT116 cells (*p* < .05; Figure 2h).

### 3.5 LINC02595 inhibits apoptosis

**Figure 3:**
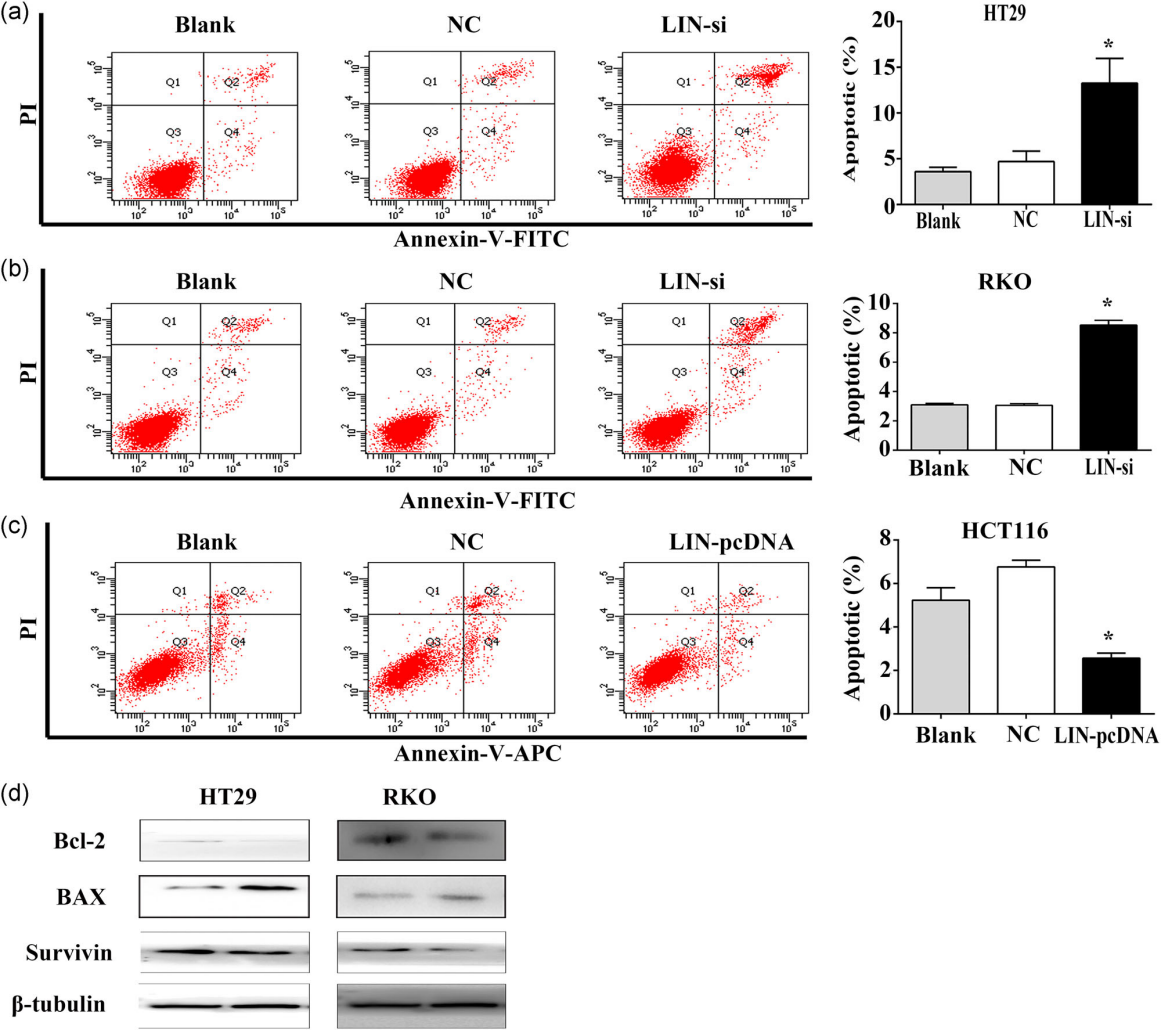
The impacts of LINC02595 on apoptosis of colorectal cancer cells. (a–c) The effect of LINC02595 on the apoptotic rate was detected in (a) HT29, (b) RKO, (c) HCT116 by flow cytometry. *p < .05. (d) Bcl2, BAX protein levels were measured by western blot in HT29 and RKO cells. FITC, fluorescein isothiocyanate; NC, negative control

To identify whether CRC cell proliferation was influenced by apoptosis, we assessed the effects of LINC02595 on apoptosis. We found that HT29 and RKO cells transfected with LINC02595 smart silencer exhibited a markedly increased rate of apoptosis compared with control cells (*p* < .05; Figure 3a,b). In contrast, LINC02595 overexpression significantly inhibited apoptosis in HCT116 cells (*p* < .05; Figure 3c). Furthermore, LINC02595 knockdown caused the downregulation of Bcl2, Survivin protein levels and upregulation of the BAX protein level of these key cell apoptosis regulators (Figure 3d).

### 3.6 LINC02595 knockdown induces G1/G0 cell cycle arrest in CRC cells

**Figure 4:**
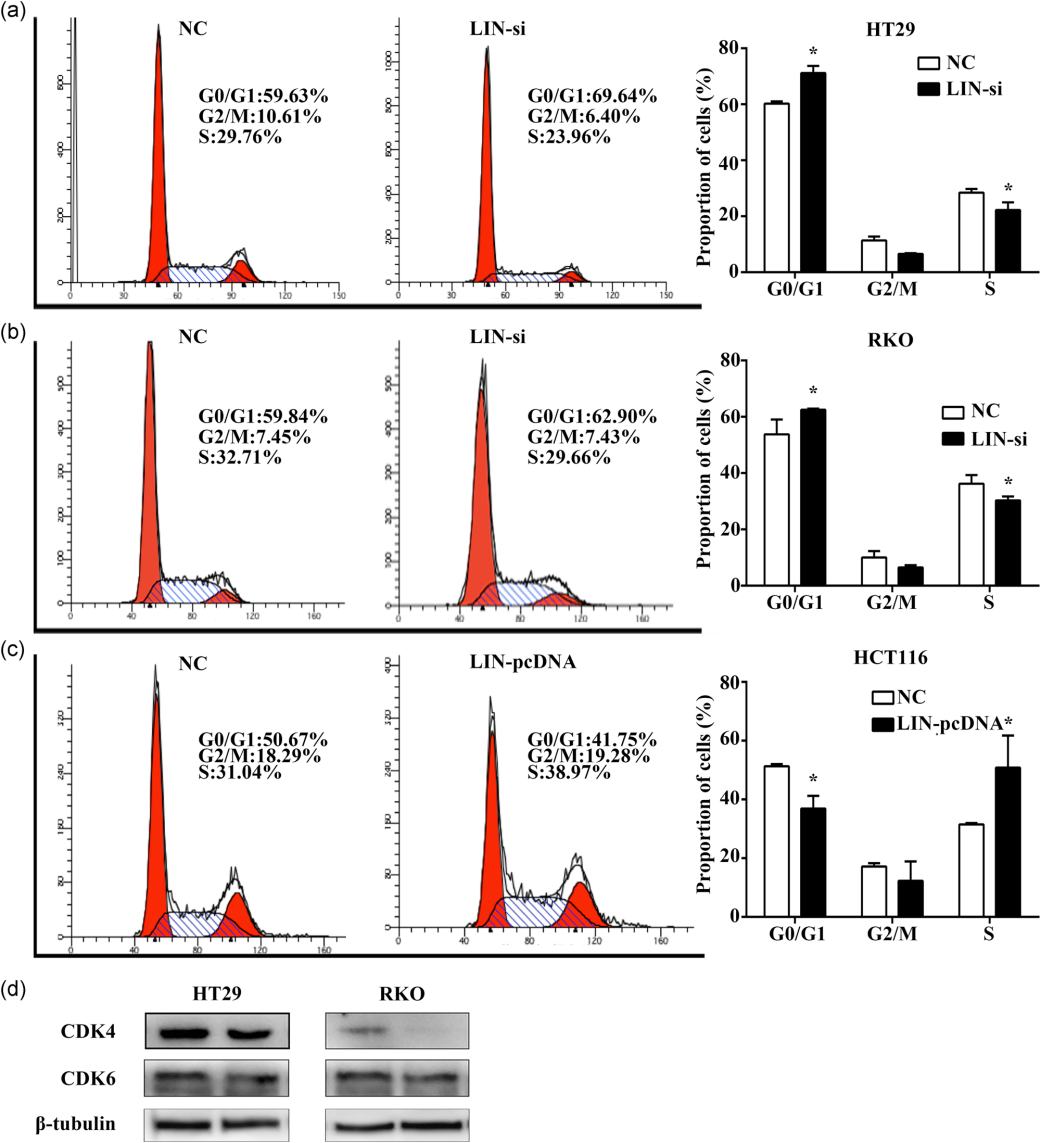
The impacts of LINC02595 on cell cycle of colorectal cancer cells. (a–c) The effect of LINC02595 on cell cycle was detected in (a) HT29, (b) RKO, (c) HCT116 by flow cytometry. *p < .05. (d) CDK4, CDK6 protein levels were measured by western blot in HT29 and RKO cells

We next evaluated the effect of LINC02595 knockdown on cell cycle progression. HT29 and RKO cells transfected with LINC02595 smart silencer had increased percentages of cells in the G1/G0 phase of the cell cycle (*p* < .05; Figure 4a,b). LINC02595 overexpression reduced the percentages of cells in this phase (*p* < .05; Figure 4c). Furthermore, LINC02595 knockdown caused the downregulation of CDK4, CDK6 protein levels of these key cell cycle regulators (Figure 4d).

### 3.7 LINC02595 knockdown inhibits the growth of CRC xenografts

**Figure 5:**
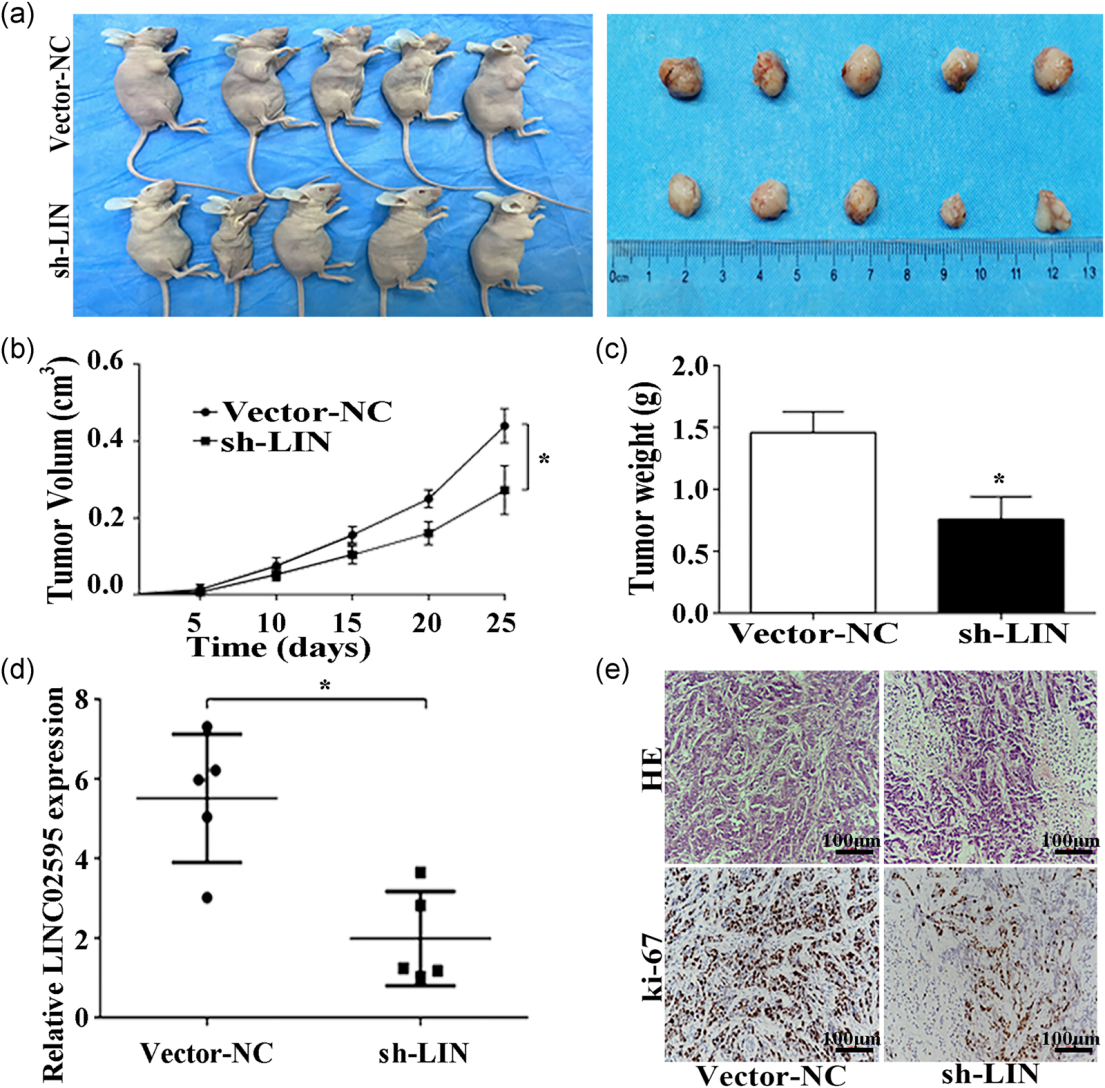
LINC02595 knockdown inhibits the growth of CRC cells in vivo. (a) Tumor formation in immunodeficient mice of the HT29 cell lines (*n* = 5 for each group). (b and c) The volume (b) and weight (c) of the xenograft tumor in two groups (*n* = 5 for each group). *p < .05. (d) The LINC02595 expression in the two groups (*n* = 5 for each group) by qRT‐PCR. *p < .05. (e) The tumor sections were evaluated by HE and IHC using antibodies against ki67 in two groups (*n* = 5 for each group). *p < .05. CRC, colorectal cancer; HE, hematoxylin and eosin; IHC, immunohistochemistry; NC, negative control; qRT‐PCR, quantitative real‐time polymerase chain reaction

To determine whether LINC02595 affected tumorigenesis in vivo, the effect of LINC02595 knockdown on the growth of CRC xenografts was evaluated. We found that sh‐LINC02595 HT29 cells formed smaller tumors in immunodeficient mice compared with the HT29‐sh‐empty control cells (*p* < .05; Figure 5a–c). As expected, these tumors expressed significantly lower levels of LINC02595 than the sh‐empty control tumors (*p* < .05; Figure 5d). Furthermore, the sh‐LINC02595 tumors had lower Ki67‐positive rates than the control tumors (*P* < 0.05; Figure 5e). Taken together, these data suggested that LINC02595 promotes tumor growth in vivo.

### 3.8 LINC02595 acts as a miR‐203b‐3p sponge

**Figure 6:**
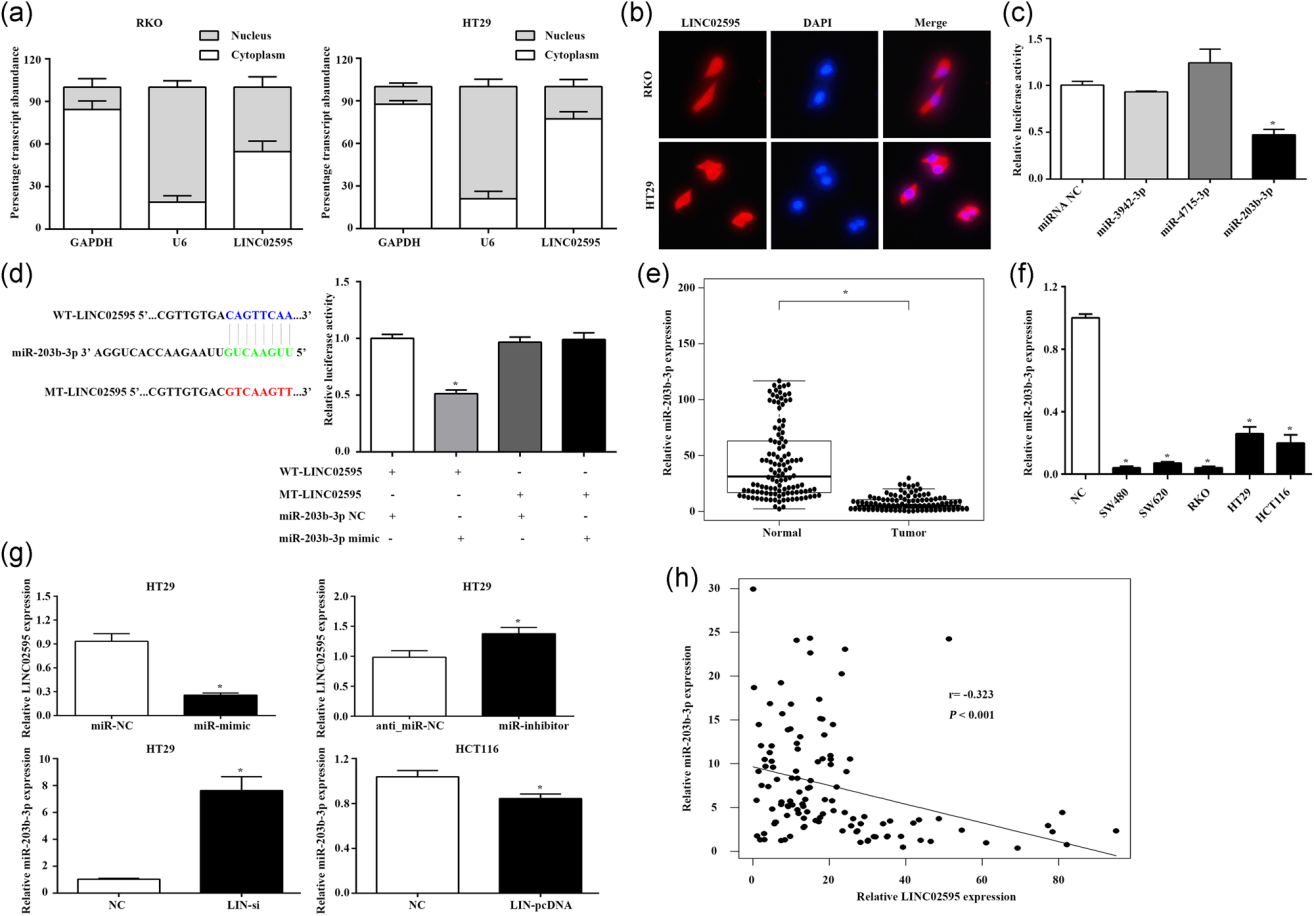
LINC02595 acts as a miR‐203b‐3p sponge. (a) The distribution of LINC02595 in RKO and HT29 cells were detected by qRT‐PCR. (b) FISH analysis of the subcellular location of LINC02595 in RKO and HT29 cells. (c) The HEK293T cell was cotransfected with wild‐type (WT) LINC02595 plasmid and miR‐3942‐3p, miR‐4715‐3p, miR‐203b‐3p mimics or NC, and the luciferase activities were measured. *p < .05. (d) Dual‐luciferase reporter assay was performed with WT‐LINC02595 and MT‐LINC02595 luciferase report vectors in HEK293T cell. The left panel was the predicted target site for miR‐203b‐3p in LINC02595. *p < .05. (e) The miR‐203b‐3p expression was detected in 116 paired CRC tissues compared with adjacent nontumor tissues. *p < .05. (f) The miR‐203b‐3p expression was detected in five CRC cell lines compared with normal colorectal epithelial tissues. *p < .05. (g) The LINC02595/miR‐203b‐3p expression was detected in miR‐mimic or miR‐inhibitor/LIN‐si or LIN‐pcDNA transfected CRC cells. *p < .05. (h) Correlation analysis between LINC02595 expression and miR‐203b‐3p expression in 116 paired tissues (*r* = −0.323; *p* < .05). CRC, colorectal cancer; FISH, fluorescence in situ hybridization; GAPDH, Glyceraldehyde 3‐phosphate dehydrogenase; miR, microRNA; NC, negative control; qRT‐PCR, quantitative real‐time polymerase chain reaction

Previous studies showed that lncRNAs could function as ceRNAs to exert their effects on cells. To determine whether LINC02595 could function as a molecular sponge for microRNA (miRNA), the distribution of LINC02595 in RKO cells and HT29 cells were determined by qRT‐PCR and FISH assay. This analysis demonstrated that LINC02595 localized to both the nucleus and cytoplasm (Figure 6a). Through FISH assay, we found that LINC02595 was mostly located in the cytoplasm (Figure 6b). All these results suggested that LINC02595 could regulate gene expression at both the transcriptional and posttranscriptional levels. Bioinformatics analysis DIANA was used to predict the miRNAs that could bind to LINC02595. We found that miR‐3942‐3p, miR‐4715‐3p, and miR‐203b‐3p generated the highest scores for binding the LINC02595 sequence.

A dual‐luciferase assay was performed to confirm the bioinformatics results. HEK293T cells were cotransfected with WT LINC02595 and miRNA mimics (miR‐3942‐3p, miR‐4715‐3p, miR‐203b‐3p) or NCs. We found that only miR‐203b‐3p significantly decreased the luciferase activity of WT LINC02595 (Figure 6c). In addition, we constructed a reporter vector in which the potential miR‐203b‐3p binding site in the sequence of LINC02595 was mutated (MT‐LINC02595), as expected, con‐transfection of the MT‐LINC02595 with miR‐203b‐3p mimic could not repress the luciferase activity (*p* < .05; Figure 6d). Furthermore, miR‐203b‐3p expression was downregulated in 116 CRC tumors tissues compared with matched control tissue (*p* = 1.254e−29; Figure 6e) and in CRC cell lines compared with normal colorectal epithelial tissues (*p* < .05; Figure 6f). Finally, LINC02595 expression was significantly abrogated by overexpression of miR‐203b‐3p and increased following miR‐203b‐3p knockdown in HT29 cells. Conversely, miR‐203b‐3p expression was induced following LINC02595 knockdown in these cells (*p* < .05; Figure 6g). Correlation analysis revealed a negative correlation between LINC02595 and miR‐203b‐3p expression in the 116 CRC tissues (*r* = −0.323; *p* < .05; Figure 6h).

### 3.9 miR‐203b‐3p mimic reverses LINC02595‐induced effects

**Figure 7:**
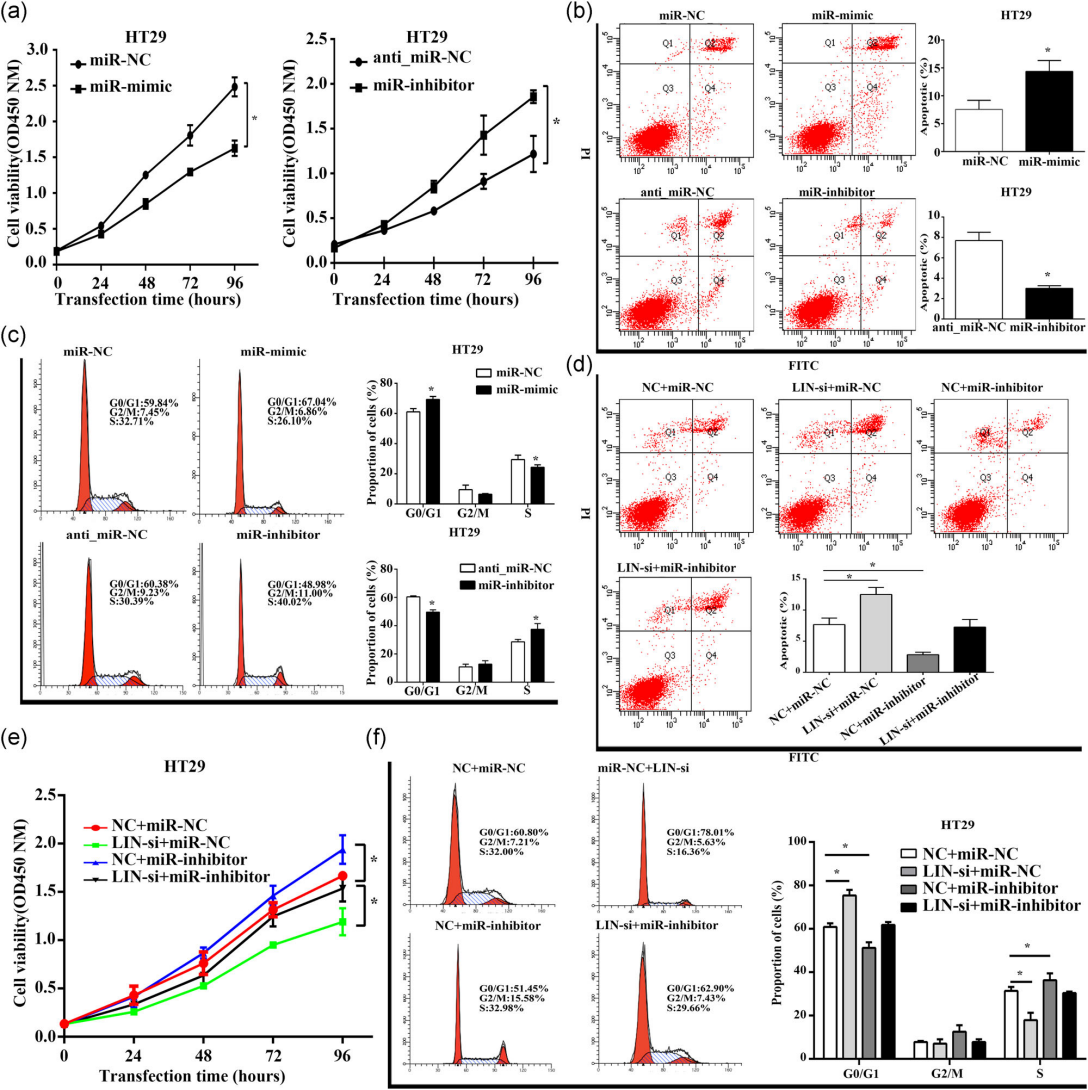
The effects of miR‐203b‐3p on CRC cells. (a) Cell proliferation was investigated by CCK‐8 in miR‐mimic and miR‐inhibitor transfected HT29. *p < .05. (b) The apoptosis ability was evaluated by flow cytometry in miR‐mimic and miR‐inhibitor transfected HT29. *p < .05. (c) The effects on cell cycle progression were evaluated by flow cytometry in miR‐mimic and miR‐inhibitor transfected HT29. *p < .05. (d) The apoptosis ability was evaluated by flow cytometry in LIN‐si, miR‐inhibitor or both transfected HT29. *p < .05. (e) Cell proliferation was investigated by CCK‐8 in LIN‐si, miR‐inhibitor or both transfected HT29. *p < .05. (f) The effects on cell cycle progression were evaluated by flow cytometry in LIN‐si, miR‐inhibitor or both transfected HT29. *p < .05. CCK‐8, cell counting kit‐8; CRC, colorectal cancer; miR, microRNA

To further understand the relationship between LINC02595 and miR‐203b‐3p, HT29 cells were transfected with an miR‐203b‐3p mimic or miR‐203b‐3p inhibitor to determine the biological function of miR‐203b‐3p in CRC. miR‐203b‐3p knockdown significantly promoted and overexpression inhibited HT29 cell proliferation (*p* < .05; Figure 7a). Furthermore, the miR‐203b‐3p mimic increased the rate of apoptosis in HT29 cells, whereas miR‐203b‐3p knockdown inhibited apoptosis (*p* < .05; Figure 7b). Moreover, the miR‐203b‐3p mimic caused G1/G0 cell cycle arrest, while the knockdown of miR‐203b‐3p significantly reduced the number of cells in G1/G0 (*p* < .05; Figure 7c). Finally, we found that miR‐203b‐3p knockdown could partially reverse the effects of LINC02595 silencing on apoptosis, proliferation, and the cell cycle (*p* < .05; Figure 7d–f). These data suggest that LINC20595 may regulate CRC proliferation, apoptosis, and the cell cycle by sponging miR‐203b‐3p.

### 3.10 LINC02595 sponges miR‐203b‐3p to regulate BCL2L1 expression in CRC

**Figure 8:**
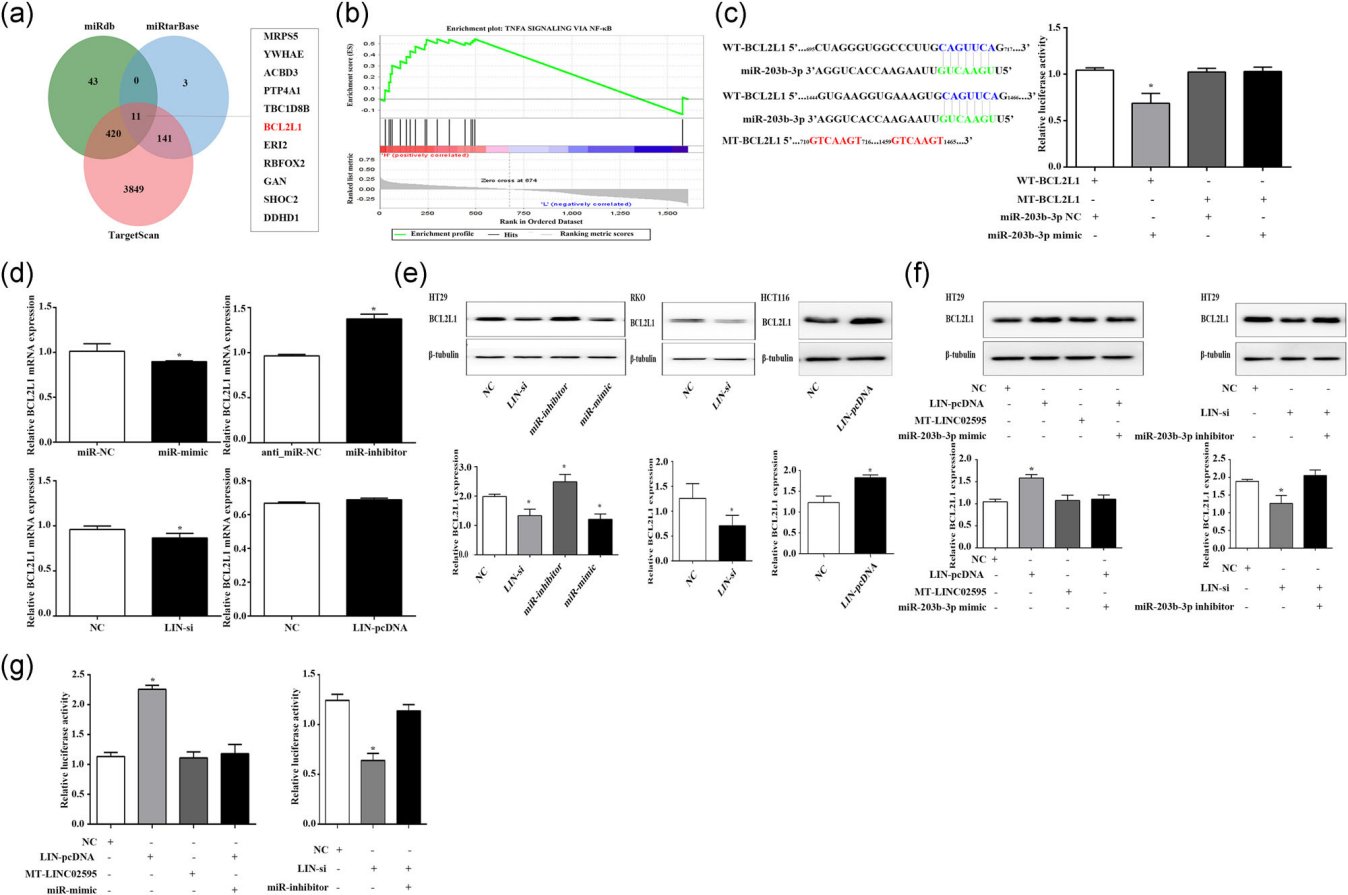
LINC02595 sponges miR‐203b‐3p to regulate BCL2L1 expression in CRC cells. (a) The potential target gene of miR‐203b‐3p was predicted by miRDB, miRTarBase, and TargetScan. (b) GSEA was performed to identify the associated biological processes of LIC02595 based on the TCGA dataset (*p* < .05). (c) Dual‐luciferase reporter assay was performed with WT‐BCL2L1 and MT‐BCL2L1 luciferase report vectors in HEK293T cell. The left panel was the predicted target site for miR‐203b‐3p in BCL2L1. *p < .05. (d) The mRNA expression of BCL2L1 was detected in CRC cells with the indicated treatment. *p < .05. (e) The protein expression of BCL2L1 was detected in miR‐mimic, miR‐inhibitor, LIN‐si, or LIN‐pcDNA transfected CRC cells. *p < .05. (f) Western blot analysis was performed in HT29 cells with the indicated treatment *p < .05. (g) Luciferase activity of Luc‐BCL2L1 reporters with the indicated treatment in HT29 cell. *p< .05. CRC, colorectal cancer; mRNA, messenger RNA; TCGA, The Cancer Genome Atlas

Three online tools (miRDB, miRTarBase, TargetScan) were used to construct the ceRNA network for the potential targets of miR‐203b‐3p. Eleven potential target genes were predicted by all three datasets. These genes were consistently upregulated (MRPS5, YWHAE, ACBD3, PTP4A1, TBC1D8B, BCL2L1, ERI2) or downregulated (RBFOX2, GAN, SHOC2, DDHD1) (Figure 8a). All their expressions were analyzed through Gene Expression Profiling Interactive Analysis (http://gepia.cancer‐pku.cn/index.html). Furthermore, we performed GSEA to identify the associated biological processes and signaling pathways based on the TCGA dataset. Patients with CRC were divided into LINC02595 high and low expression groups based on the median value of LINC02595 expression. The GSEA hallmark of LINC02595 between these two groups showed enrichment for the nuclear factor‐κB (NF‐κB) signaling pathways (*p* < .05; Figure 8b). These findings suggested that LINC02595 regulates cell proliferation through NF‐κB signaling to regulate downstream target genes. Of the 11 potential target genes, only BCL2L1 was related to NF‐κB signaling.

Aakko et al. (2019) previously demonstrated that BCL2L1 has a binding site for miR‐203b‐3p in breast cancer. Thus, BCL2L1 was chosen as a target for our further studies to understand whether BCL2L1 and miR‐203b‐3p have the same mechanism in CRC. A dual‐luciferase assay was performed to confirm the bioinformatics results. HEK293T cells were cotransfected with WT‐BCL2L1 and miR‐203b‐3p mimic or NC. We found that miR‐203b‐3p significantly decreased the luciferase activity of WT‐BCL2L1, but not the MT‐BCL2L1 (*p* < .05; Figure 8c). In addition, miR‐203b‐3p overexpression significantly reduced BCL2L1 mRNA and protein levels in HT29 cells (*p* < .05; Figure 8d,e). In contrast, miR‐203b‐3p knockdown promoted BCL2L1 mRNA and protein expression (*p* < .05; Figure 8d,e). As LINC02595 could function as a ceRNA for miR‐203b‐3p, we investigated whether LINC02595 could indirectly regulate the expression of BCL2L1. We found that LINC02595 knockdown reduced BCL2L1 mRNA and protein expression, whereas LINC02595 overexpression promoted BCL2L1 protein expression only in CRC cells (*p* < .05; Figure 8d,e). To further demonstrate that LINC02595 could regulate BCL2L1 expression through sponging miR‐203b‐3p, western blot analysis assays showed that transfection of the miR‐203b‐3p mimic abolished BCL2L1 increase in LINC02595 upregulated cells (*p* < .05; Figure 8f). Furthermore, the miR‐203b‐3p knockdown could rescue the BCL2L1 protein level downregulation induced by LINC02595 (*p *< .05; Figure 8f). Finally, we performed dual‐luciferase reporter assays in HT29 cells, the results showed that the transfection of WT‐LINC02595 plasmids but not the MT‐LINC02595 plasmids could increase Luc‐BCL2L1 luciferase activity, and the transfection of miR‐203b‐3p mimic could reduce the luciferase activity increase induced by overexpression of LINC02595, whereas LINC02595 knockdown reduced Luc‐BCL2L1 luciferase activity significantly, and transfection of the miR‐203b‐3p inhibitor antagonized this decrease (*p* < .05; Figure 8g). All these results showed that LINC02595 could regulate BCL2L1 expression by sponging miR‐203b‐3p in CRC.

### 3.11 BCL2L1 is upregulated in CRC

**Figure 9:**
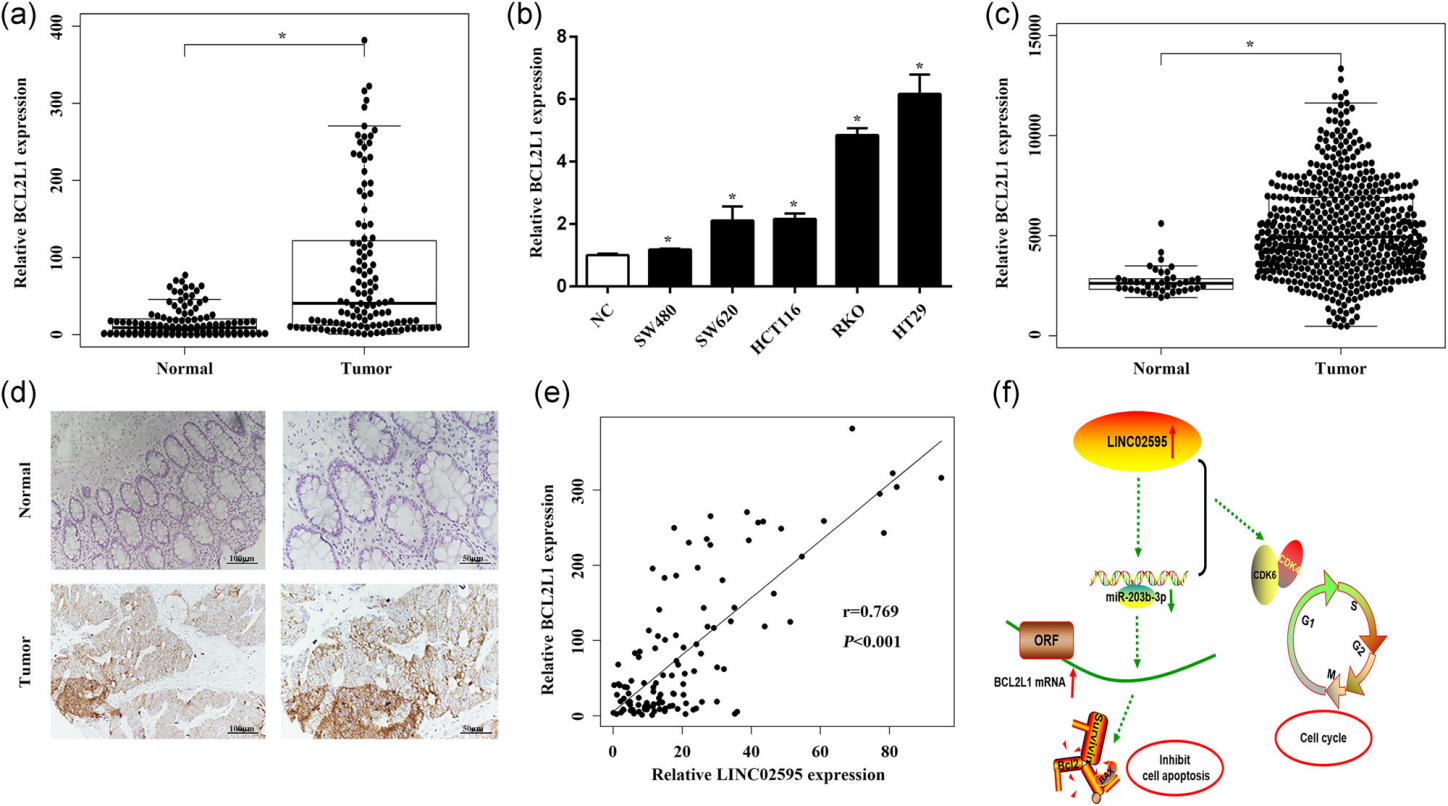
The effect of BCL2L1 in CRC. (a) The mRNA expression of BCL2L1 was detected in 116 paired CRC tumor tissues compared with adjacent nontumor tissues. *p < .05. (b) The mRNA expression of BCL2L1 was detected in five CRC cell lines compared with normal colorectal epithelial tissues. *p < .05. (c) The mRNA expression of BCL2L1 was analyzed based on TCGA transcriptome profiling. *p < .05. (d) The protein expression of BCL2L1 was evaluated by IHC in CRC tumor tissues compared with adjacent nontumor tissues. *p < .05. (e) Correlation analysis between LINC02595 and BCL2L1 in 116 paired tissues (*r* = 0.769; *p* < .05). (f) Schematic representation of LINC02595 in CRC. LINC02595 could sponge miR‐203b‐3p and regulate BCL2L1 expression. CRC, colorectal cancer; IHC, immunohistochemistry; mRNA, messenger RNA; TCGA, The Cancer Genome Atlas

To understand the function of BCL2L1 in CRC progression, we measured BCL2L1 expression levels in 116 paired CRC and normal tissues and CRC cell lines by qRT‐PCR. We found that BCL2L1 expression levels were significantly upregulated in the CRC tumor tissues and cell lines compared with the adjacent nontumor tissues (*p* < .05; Figure 9a,b). These results were confirmed by TCGA transcriptome profiling analysis (*p* < .05; Figure 9c). Furthermore, IHC showed that BCL2L1 protein levels were also upregulated in the CRC tumors compared with the adjacent nontumor tissue (*p* < .05; Figure 9d). Correlation analysis revealed a positive correlation between the LINC02595 and BCL2L1 expression levels in the 116 CRC tissues (*r* = 0.769; *p* = 6.248e−24; Figure 9e) and the TCGA dataset (*r* = 0.231; *p* = 5.325e−08; Figure S1d). Together, these results identified an important LINC02595/miR‐203b‐3p/BCL2L1 regulatory axis in CRC (Figure 9f).

## 4 DISCUSSION

CRC is the third leading cause of cancer mortality. Despite significant advances in the diagnosis and treatment of cancer, progress in improving the OS of patients with CRC has been slow. lncRNAs have been the focus of the search for new biomarkers to improve the prognosis of CRC patients due to the accumulating evidence that lncRNAs have an essential regulatory function in tumorigenesis (A. Liu, Liu, & Lu, 2019; P. F. Zhang et al., 2019). In recent years, Xian et al. (Xian & Zhao, 2019) found that KCNQ1OT1 was upregulated in methotrexate‐resistant cells and promoted the methotrexate resistance of CRC cells by regulating miR‐760/PPP1R1B signaling. G. Li, Wang, Wang, Xu, and Zhang (2018) showed that LINC00312 was downregulated in CRC tissues and cell lines, and cell growth, migration, and invasion could be suppressed by regulating miR‐21. In this study, we demonstrated for the first time the presence of LINC02595 and the underlying molecular mechanism of its function in CRC. Specifically, we established that LINC02595 could function as a ceRNA to regulate the miR‐203b‐3p/BCL2L1 regulatory axis and activate the NF‐κB signaling pathway.

Although colonoscopy is the gold standard for CRC diagnosis, it is time‐consuming and requires a specialist for diagnosis. Therefore, the identification of tumor biomarkers has significant consequences in the diagnosis of this disease. In recent years, there has been an increasing number of blood‐based miRNAs studies; however, the evaluation of circulating lncRNAs as tumor biomarkers in plasma is still in its infancy. In our study, we investigated the diagnostic potential of LINC02595 in plasma using ROC analysis and determined a cut‐off value of 1.507 and AUC of 0.781. As we demonstrated that LINC02595 is upregulated in both CRC tissues and plasma as well as IBD patient tissues, LINC02595 might represent an independent biomarker for the early detection of CRC.

It is widely accepted that lncRNAs can act as ceRNAs for miRNAs to sequester them from their target mRNAs, resulting in the upregulation of mRNA expression. Thus, we used the online prediction tool (http://carolina.imis.athena‐innovation.gr/diana_tools/web/index.php) to identify potential miRNAs that interact with LINC02595. Among candidate miRNAs, miR‐203b‐3p was selected due to its tumor‐suppressing role in multiple cancers, such as lung adenocarcinoma (Ge et al., 2019; Jiang et al., 2019), gastric cancer (Liu et al., 2016), ovarian cancer (Wang et al., 2018), and hepatocellular carcinoma (Furuta et al., 2010). However, the pivotal role of miR‐203b‐3p was not previously studied in‐depth in CRC. Previous studies showed that miR‐203b‐3p was downregulated in CRC tissues and cell lines and could inhibit cell proliferation, invasion, and migration (Chiang et al., 2011; Z. Li et al., 2015; Ma et al., 2018). Hur et al. (2017) demonstrated that miR‐203 expression was upregulated in liver metastasis compared with matched primary CRC tumors as well as in CRC serum. There are also reports demonstrating that miR‐203 expression levels correlated with chemotherapy resistance (Li, Chen, Zhao, Kong, & Zhang, 2011; T. Li, Gao, & Zhang, 2015). In this study, we found that miR‐203b‐3p was downregulated in CRC cell lines and tissues compared with adjacent nontumor tissues, and overexpression of miR‐203b‐3p inhibited cell proliferation, increased the rate of apoptosis, and caused G1/G0 cell cycle arrest. Furthermore, we found that the inhibition of these effects by the LINC02595 smart silencer could be rescued by a miR‐203b‐3p inhibitor. We also found a negative correlation between the expression levels of LINC02595 and miR‐203b‐3p in 116 CRC samples. However, miR‐203b‐3p was upregulated in CRC samples from the TCGA dataset, which was in contrast with our experimental conclusion.

lncRNAs act as ceRNA through the inhibition of its miRNA target gene. In our study, we identified BCL2L1 as the target gene of miR‐203b‐3p using bioinformatic tools (miRDB, miRTarBase, TargetScan). GSEA analysis showed that high expression of LINC02595 was enriched for the NF‐κB signaling pathway, and BCL2L1 is one of the most important components of NF‐κB signaling. In 1986, NF‐κB was found to interact with the 11‐base pair sequence in the immunoglobulin light‐chain enhancer of B cells. Multiple papers demonstrated that it was a key factor involved in cell proliferation, inflammation, cell death, and cell survival. Two general NF‐κB signaling pathways, which include the classical and alternative pathways were shown to correlate with tumor progression. The targets of NF‐κB signaling involving the classical NF‐κB signaling pathway include antiapoptotic proteins (e.g., BclxL, BCL2) and pro‐proliferative proteins (e.g., cyclin D1, MYC; Karin, 2006). X. Liu et al. (2019) found that the adaptively expressed endogenous epidermal growth factor could activate the cyclin D1/P53/PARP signaling pathway in pancreatic cancer cells. Aakko et al. (2019) also demonstrated that BCL2L1 has a miR‐203b‐3p binding site in breast cancer. BCL2L1 is a member of the Bcl2 protein family that regulates mitochondrial‐mediated apoptosis by regulating the release of proapoptotic factors from the mitochondria (Chen et al., 2005). BCL2L1 has been identified as an important survival factor in many tumor types (Obasi et al., 2018; Zhang et al., 2015; P. F. Zhang et al., 2019). In this study, we demonstrated that BCL2L1 is overexpressed in CRC compared with adjacent nontumor tissue, and a positive correlation existed between the expression levels of LINC02595 and BCL2L1 in 116 CRC tissues and the TCGA dataset.

Our study systematically analyzed the correlation of LINC02595, miR‐203b‐3p, and BCL2L1 in the modulation of CRC progression. We also demonstrated through the analysis of a large number of samples that LINC02595 might be a potential target for diagnosis and treatment of CRC. However, there are still several limitations to our study. First, our cohort study lacks survival time information, which affected our survival analysis based on LINC02595 expression. Furthermore, the plasma sample size presented in our study was confined to one ethnicity. Therefore, the study results might not apply to the general population. Finally, we only analyzed the role of LINC02595 in the progression of CRC as a ceRNA for miR‐203b‐3p to regulate the expression of BCL2L1. Analysis of its role in CDK6, CDK4, BCL2, Survivin, and BAX protein expression is currently in progress. How LINC02595 exerts its effect on the NF‐κB signaling pathway requires further study.

## CONFLICT OF INTERESTS

The authors declare that there are no conflict of interests.

## AUTHOR CONTRIBUTIONS

All authors equally contributed to this work.

## ETHICS STATEMENT

The study protocol was approved by the Research Ethics Committee of The First Affiliated Hospital of China Medical University. All patients provided written informed consent for participation in this study.

## Supporting information

Supporting information

Supporting information

Supporting information

Supporting information

## Data Availability

All data generated or analyzed in this study are included in this published article.
