# Supplementary figures and images for: LINC02595 promotes tumor progression in colorectal cancer by inhibiting miR‐203b‐3p activity and facilitating BCL2L1 expression
Source: J Cell Physiol. 2020 Feb 16;235(10):7449–64. doi: 10.1002/jcp.29650 (PMC7496558; doi:10.1002/jcp.29650)

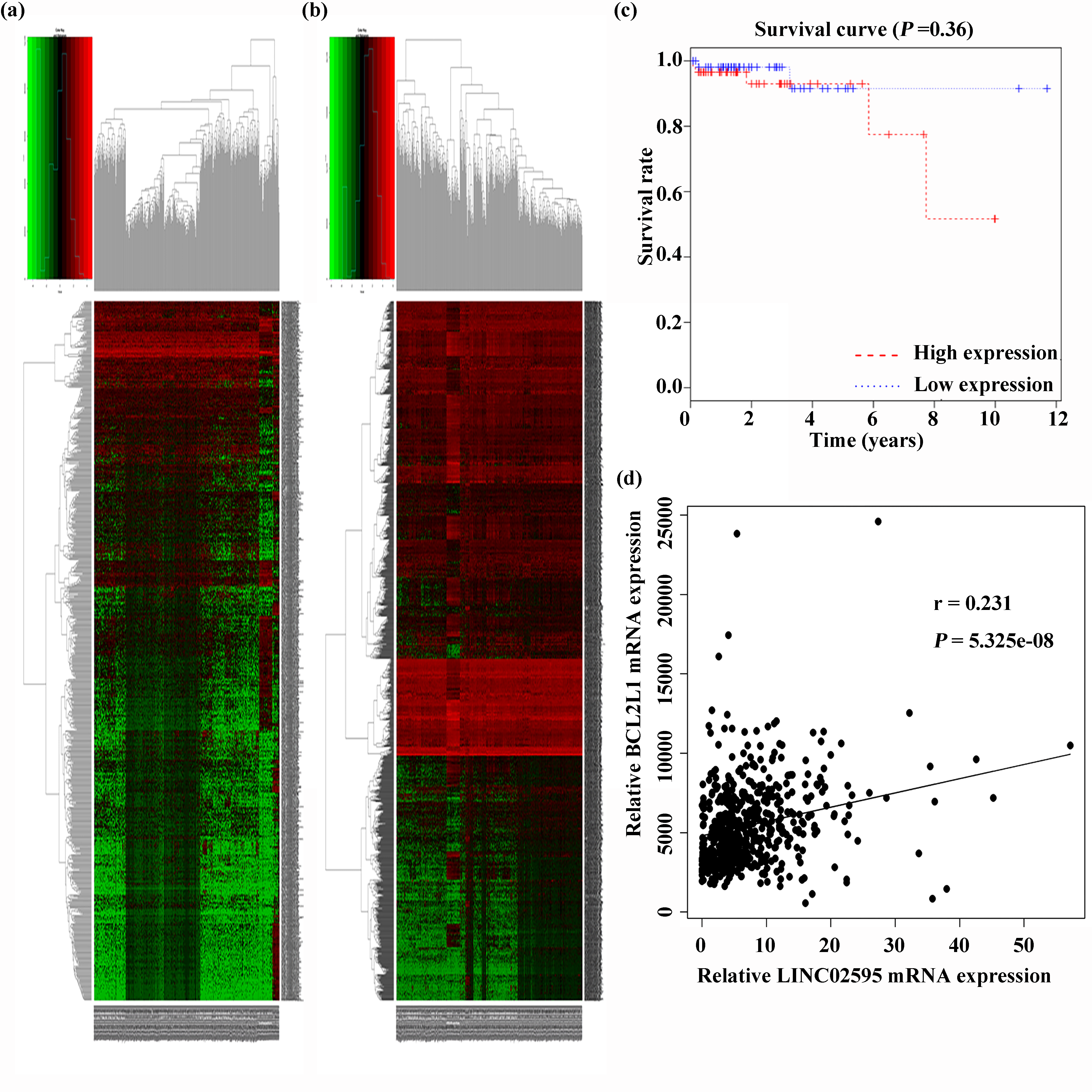

Supplement: Supplementary file 1 — Supporting information [file JCP-235-7449-s001.tif]
