# Supplementary material for: LINC02595 promotes tumor progression in colorectal cancer by inhibiting miR‐203b‐3p activity and facilitating BCL2L1 expression
Source: J Cell Physiol. 2020 Feb 16;235(10):7449–64. doi: 10.1002/jcp.29650 (PMC7496558; doi:10.1002/jcp.29650)
Supplement: Supplementary file 2 — Supporting information [file JCP-235-7449-s002.docx]

Table S1 The first 15 upregulated and downregulated lncRNA transcripts in microarray

| ID | Regulation | Fold change | *P* value |
| --- | --- | --- | --- |
| ENST00000561864.1 | up | 23.35148238 | 0.001917413 |
| XR_108886.3 | up | 16.93536018 | 0.001391076 |
| ENST00000500112.1 | up | 14.20293763 | 0.001183682 |
| ENST00000419422.1 | up | 13.60426539 | 0.002825793 |
| ENST00000602761.1 | up | 12.44606998 | 0.009106789 |
| ENST00000437781.1 | up | 12.21613146 | 0.00618077 |
| ENST00000447298.1 | up | 9.872537096 | 0.005056757 |
| TCONS_00014191 | up | 9.779919785 | 0.017538194 |
| ENST00000414790.1 | up | 9.736971877 | 0.004289948 |
| ENST00000429700.1 | up | 9.240787746 | 0.02594489 |
| ENST00000456532.1 | up | 9.068814828 | 0.00027043 |
| TCONS_00015168 | up | 8.893084227 | 0.004170451 |
| ENST00000521586.1 | up | 8.54294018 | 0.005211174 |
| ENST00000427798.1 | up | 8.208992276 | 0.011856653 |
| ENST00000544553.1 | up | 7.599453362 | 0.006032456 |
| uc.217+ | down | 28.08411927 | 0.001419577 |
| ENST00000497872.2 | down | 14.2164372 | 0.017713116 |
| ENST00000506514.1 | down | 14.20444811 | 0.002509867 |
| ASO1844 | down | 14.15365832 | 0.002284565 |
| TCONS_00028105 | down | 14.0751825 | 0.01025426 |
| uc022axe.1 | down | 14.05103981 | 0.018601647 |
| ENST00000591283.1 | down | 14.03575661 | 0.002672382 |
| ENST00000596996.1 | down | 13.85497334 | 0.015292863 |
| ENST00000572964.1 | down | 13.56753412 | 0.006808164 |
| ENST00000421632.1 | down | 13.06146879 | 0.015701222 |
| NR_026542.1 | down | 12.50157151 | 0.002734412 |
| ENST00000422420.1 | down | 12.25588173 | 0.013418821 |
| TCONS_00008010 | down | 12.09342747 | 0.001855295 |
| ENST00000416200.1 | down | 10.21323974 | 0.000279853 |
| HIT000392999 | down | 10.11594753 | 0.017349789 |
