# Supplementary material for: LINC02595 promotes tumor progression in colorectal cancer by inhibiting miR‐203b‐3p activity and facilitating BCL2L1 expression
Source: J Cell Physiol. 2020 Feb 16;235(10):7449–64. doi: 10.1002/jcp.29650 (PMC7496558; doi:10.1002/jcp.29650)
Supplement: Supplementary file 3 — Supporting information [file JCP-235-7449-s003.docx]

Table S2 The first 15 upregulated and downregulated mRNA transcripts in microarray

| ID | Regulation | Fold change | *P* value |
| --- | --- | --- | --- |
| ENST00000260227 | up | 121.6482552 | 0.000151673 |
| ENST00000233735 | up | 81.61951274 | 0.017741592 |
| ENST00000488355 | up | 78.00741528 | 0.000321295 |
| ENST00000304725 | up | 58.29034488 | 0.000837811 |
| ENST00000398402 | up | 48.46500228 | 0.000683138 |
| ENST00000599690 | up | 37.43715598 | 0.032517126 |
| ENST00000296839 | up | 35.94445425 | 0.001954736 |
| ENST00000462312 | up | 34.50183257 | 0.00179923 |
| ENST00000299855 | up | 32.30381622 | 0.009004792 |
| ENST00000315274 | up | 30.16469694 | 0.013611643 |
| ENST00000352040 | up | 20.88325415 | 0.00185641 |
| ENST00000429102 | up | 20.4851984 | 0.002279316 |
| ENST00000322507 | up | 20.37191593 | 0.000894715 |
| ENST00000395761 | up | 20.11914086 | 0.009816137 |
| ENST00000188790 | up | 19.53330575 | 0.003742267 |
| ENST00000523022 | down | 143.8527979 | 0.01097354 |
| ENST00000375497 | down | 71.50469773 | 0.003194952 |
| ENST00000287641 | down | 60.08515286 | 0.001967549 |
| ENST00000613166 | down | 54.86468892 | 0.004558079 |
| ENST00000234701 | down | 48.7877124 | 0.017379976 |
| ENST00000606252 | down | 47.9750965 | 0.001603517 |
| ENST00000618265 | down | 47.34352423 | 0.002230076 |
| ENST00000304526 | down | 46.66623397 | 0.009375444 |
| ENST00000417164 | down | 32.66271448 | 0.002264948 |
| ENST00000357001 | down | 30.25597849 | 0.005879232 |
| ENST00000606252 | down | 29.75863958 | 0.003405589 |
| ENST00000340010 | down | 27.72614004 | 0.01787136 |
| ENST00000400752 | down | 27.18434595 | 0.025528077 |
| ENST00000590317 | down | 26.92347302 | 0.012442693 |
| ENST00000264382 | down | 24.11553143 | 0.017653957 |
