# Supplementary material for: LINC02595 promotes tumor progression in colorectal cancer by inhibiting miR‐203b‐3p activity and facilitating BCL2L1 expression
Source: J Cell Physiol. 2020 Feb 16;235(10):7449–64. doi: 10.1002/jcp.29650 (PMC7496558; doi:10.1002/jcp.29650)
Supplement: Supplementary file 4 — Supporting information [file JCP-235-7449-s004.docx]

Table S3 The clinic pathological factors of 506 CRC patients

| Characteristics | Number of cases | LINC02595 expression | | *P* value^a^ |
| --- | --- | --- | --- | --- |
|  |  | Low (n = 58) | High (n = 58) |  |
| Age(year) |  |  |  | 0.922 |
| <60 | 147 | 73 | 74 |  |
| ≥60 | 359 | 180 | 179 |  |
| Gender |  |  |  |  |
| Female | 230 | 107 | 123 | 0.153 |
| Male | 276 | 146 | 130 |  |
| Tumor invasion depth |  |  |  |  |
| T1+T2 | 106 | 52 | 54 | 0.827 |
| T3+T4 | 400 | 201 | 199 |  |
| Lymph node metastasis |  |  |  |  |
| N0 | 295 | 145 | 150 | 0.718 |
| N1+N2 | 211 | 108 | 103 |  |
| Distant metastasis |  |  |  |  |
| M0 | 382 | 191 | 191 | 0.541 |
| M1+M2 | 124 | 62 | 62 |  |
| Cancer Type |  |  |  |  |
| Rectal cancer | 89 | 51 | 38 | 0.161 |
| Colon cancer | 417 | 202 | 215 |  |

^a^Statistical significant results (in italics)
